# Synapse-Centric Mapping of Cortical Models to the SpiNNaker Neuromorphic Architecture

**DOI:** 10.3389/fnins.2016.00420

**Published:** 2016-09-14

**Authors:** James C. Knight, Steve B. Furber

**Affiliations:** Advanced Processor Technologies Group, School of Computer Science, University of ManchesterManchester, UK

**Keywords:** SpiNNaker, learning, plasticity, digital neuromorphic hardware, event-driven simulation, cortical network, BCPNN

## Abstract

While the adult human brain has approximately 8.8 × 10^10^ neurons, this number is dwarfed by its 1 × 10^15^ synapses. From the point of view of neuromorphic engineering and neural simulation in general this makes the simulation of these synapses a particularly complex problem. SpiNNaker is a digital, neuromorphic architecture designed for simulating large-scale spiking neural networks at speeds close to biological real-time. Current solutions for simulating spiking neural networks on SpiNNaker are heavily inspired by work on distributed high-performance computing. However, while SpiNNaker shares many characteristics with such distributed systems, its component nodes have much more limited resources and, as the system lacks global synchronization, the computation performed on each node must complete within a fixed time step. We first analyze the performance of the current SpiNNaker neural simulation software and identify several problems that occur when it is used to simulate networks of the type often used to model the cortex which contain large numbers of sparsely connected synapses. We then present a new, more flexible approach for mapping the simulation of such networks to SpiNNaker which solves many of these problems. Finally we analyze the performance of our new approach using both benchmarks, designed to represent cortical connectivity, and larger, functional cortical models. In a benchmark network where neurons receive input from 8000 STDP synapses, our new approach allows 4× more neurons to be simulated on each SpiNNaker core than has been previously possible. We also demonstrate that the largest plastic neural network previously simulated on neuromorphic hardware can be run in real time using our new approach: double the speed that was previously achieved. Additionally this network contains two types of plastic synapse which previously had to be trained separately but, using our new approach, can be trained simultaneously.

## 1. Introduction

Various types of hardware have been used as the basis for large-scale neuromorphic systems: NeuroGrid (Benjamin et al., 2014) and BrainScaleS (Schemmel et al., 2010) use custom analog hardware; True North (Merolla et al., 2014) uses custom digital hardware and SpiNNaker (Furber et al., 2014) uses software programmable ARM processors.

The design of SpiNNaker was based on the assumption that each ARM processing core would be responsible for simulating 1000 spiking neurons (Jin et al., 2008). Each of these neurons was expected to have around 1000 synaptic inputs each receiving spikes at an average rate of 10 Hz and, within these constraints, large-scale cortical models with up to 50 × 10^6^ neurons have already been successfully simulated on SpiNNaker (Sharp et al., 2014).

However over recent years it has become clear that larger, more realistic brain models are likely to break these assumptions. For the purpose of this paper we concentrate on models of the cortex where anatomical data (Beaulieu and Colonnier, 1989; Pakkenberg et al., 2003; Braitenberg and Schüz, 2013) suggests that, across species, cortical neurons received an average of around 8000 synaptic inputs. In Sections 2.1, 2.2 we will analyze the performance of the current SpiNNaker neural simulation kernel and how it is impacted by these higher degrees of connectivity.

Neuromorphic systems built from custom hardware have the potential to simulate large neural models using many orders of magnitude less power than software programmable systems such as SpiNNaker. However, in these systems, a fixed number of circuits for simulating individual neurons and synapses are often directly cast into hardware. Therefore, coping with higher degrees of connectivity than these systems' designers intended is, at worst, impossible and, at best, can only be achieved by “borrowing” synapses from other neurons leaving some neuron circuits without any synapses to provide them with input. For example as Schemmel et al. (2010) discuss, by “borrowing” multiple rows of 224 synapses from other neurons, each neuron on the BrainScaleS system can receive up to 14,336 synaptic inputs.

Because the basic computational unit of a SpiNNaker system is a general-purpose processor, exactly how simulations of spiking neural networks are mapped to the system is defined, largely, in software. For example Mundy et al. (2015) used this flexibility to map spiking neural networks—specified using the Neural Engineering Framework (Eliasmith and Anderson, 2004)—to SpiNNaker in a novel manner which removes the need to simulate individual synapses. In this paper we take a very different approach and, in Section 2.3, present a novel technique for mapping spiking neural networks to SpiNNaker which we call “synapse-centric mapping.” In Section 3 we demonstrate the advantages of this new approach both in benchmarks and in several large-scale cortical network models. Finally, in Section 4, we discuss how this approach has the potential to enable the simulation of multi-compartmental neuron models on SpiNNaker and its applicability to current and future distributed computing platforms.

## 2. Materials and methods

### 2.1. Simulating spiking neural networks on SpiNNaker

**Figure 1:**
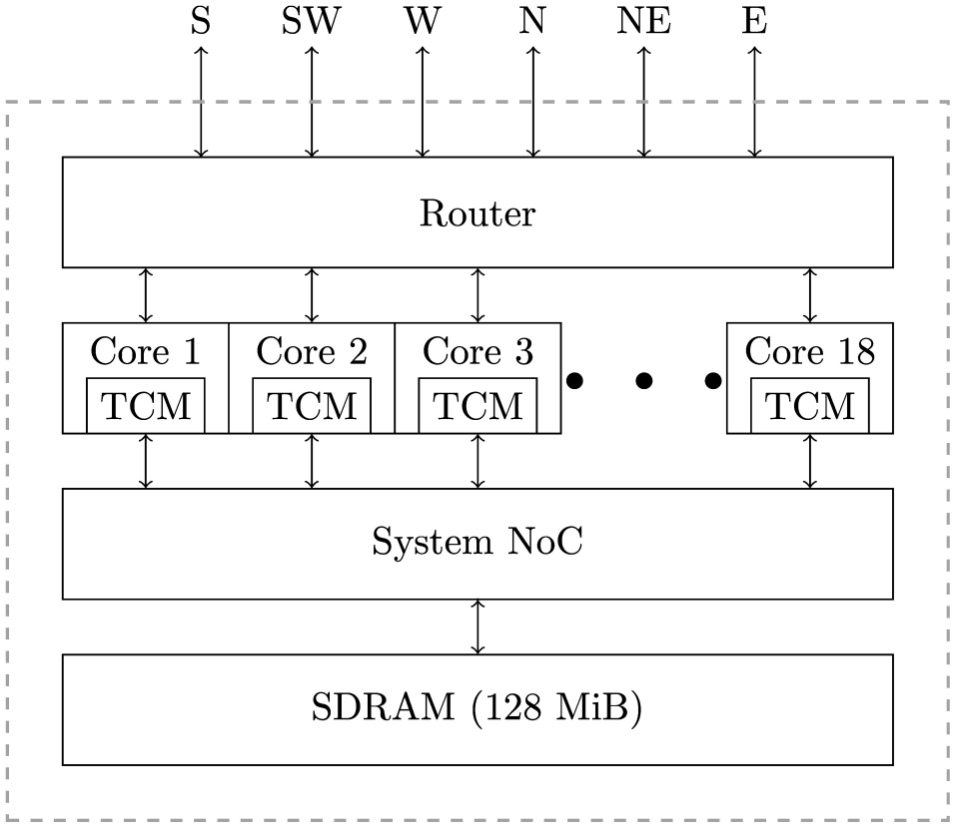
**The basic architecture of a SpiNNaker chip**.

SpiNNaker is a massively parallel architecture designed primarily for the simulation of spiking neural networks. The SpiNNaker architecture can be used to build systems, ranging in size from single boards to room-size machines, but all using the same basic building block: the SpiNNaker chip (Furber et al., 2014). As shown in Figure 1, a SpiNNaker chip contains 18, 200 MHz ARM cores, each equipped with two small tightly-coupled memories (TCM): 32 KiB for instructions and 64 KiB for data. The cores within a chip are then connected to each other, 128 MiB of external SDRAM and a multicast router using a network-on-chip (NoC) known as the “System NoC.” Every chip's router is then connected to the routers in the six immediate neighboring chips using a second NoC known as the “Communications NoC.”

**Figure 2:**
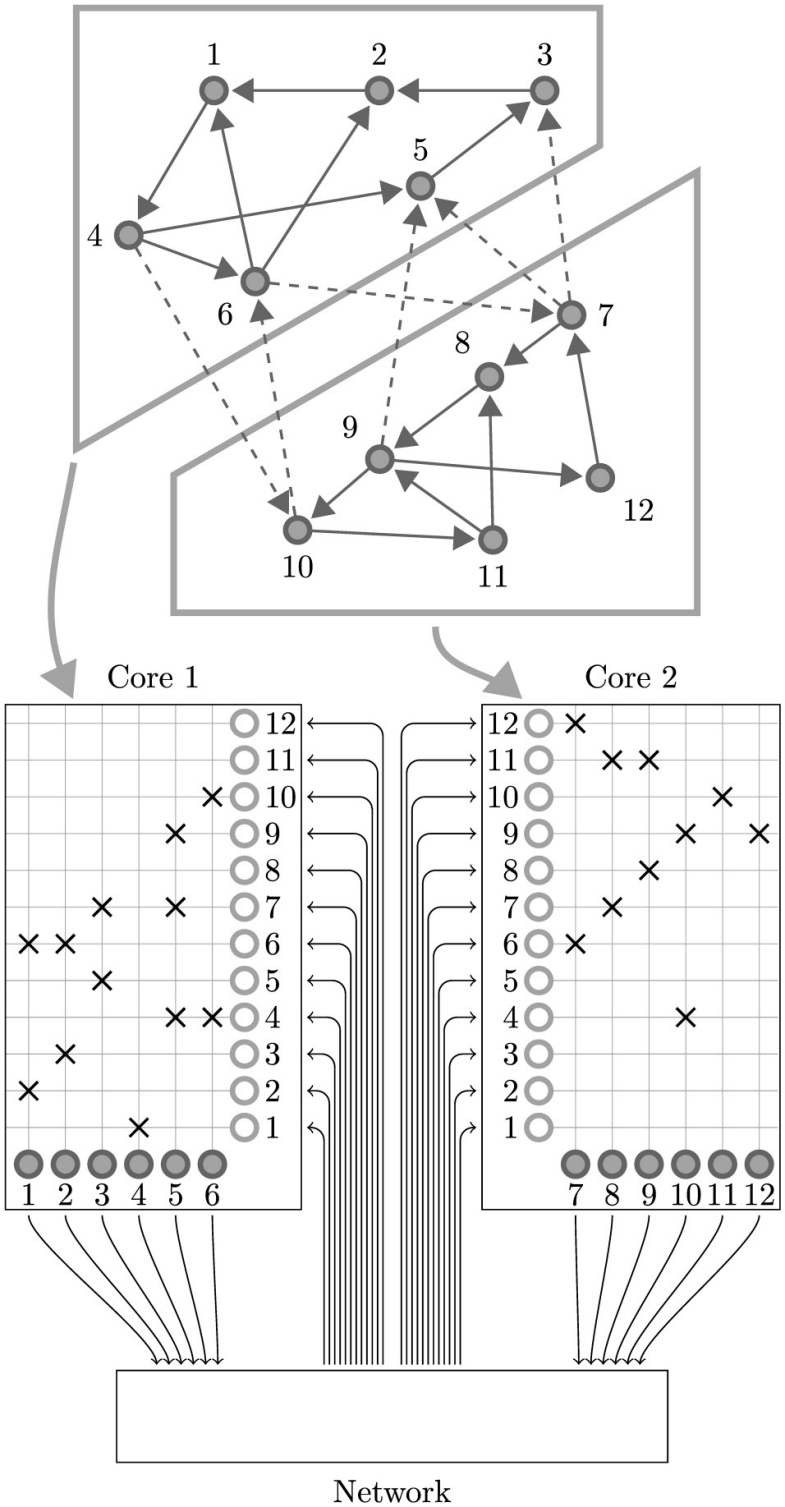
**Standard mapping of a spiking neural network to SpiNNaker**. An example network consisting of 12 neurons is distributed between two SpiNNaker cores. The synaptic matrix is split vertically and its columns are distributed between the two cores responsible for simulating the corresponding postsynaptic neurons (filled circles). Both cores contain synaptic matrix rows corresponding to all 12 presynaptic neurons (non-filled circles). The SpiNNaker router routes spikes from firing neurons (filled circles) to the cores responsible for simulating the neurons these spikes target.

While SpiNNaker has a somewhat unusual memory hierarchy, the lack of global shared memory means that many of the problems related to simulating large spiking neural networks on a SpiNNaker system are shared with more typical distributed computer systems. On this basis the SpiNNaker neural simulator follows a very similar approach to that developed by Morrison et al. (2005) and Kunkel et al. (2012) for mapping large spiking neural networks onto large distributed systems. Figure 2 illustrates this mapping in the context of SpiNNaker with each processing core being responsible for simulating a collection of neurons and their afferent synapses. The neurons are simulated using a time-driven approach and their state is held in the tightly-coupled data memory. Each neuron is uniquely identified by a 32 bit ID and, if a simulation step results in a spike, a packet containing this ID is sent to the SpiNNaker router. These “spike” packets are then routed across the network fabric to all the cores on which neurons with afferent synaptic connections from the spiking neuron are simulated.

However because of the large number of synapses and the relatively low firing rate of single neurons, the synapses are simulated in the event-driven manner discussed by Morrison et al. (2005) only getting updated when they transfer a spike. On SpiNNaker this event-driven approach is also advantageous as, due to the sheer number of synapses, per-synapse data such as synaptic weights must be stored in the off-chip SDRAM which has insufficient bandwidth (Painkras et al., 2013) to transfer every synapse's parameters each simulation time step. Instead, on receipt of a “spike” packet, cores initiate a DMA transfer to fetch the row of the connectivity matrix associated with the firing neuron from SDRAM (Sharp et al., 2011). Each of these rows describes the synaptic connections between a presynaptic neuron and the postsynaptic neurons simulated on the core. Once a row is retrieved, the synaptic weights it contains are inserted into an input ring-buffer, where they remain until any synaptic delay has elapsed and they are added to the correct neuron's input current. Because of the large number of synapses the performance of the synaptic row processing stage is critical and, as such, has been significantly optimized since Sharp and Furber (2013) measured its performance at 32 clock cycles per synapse. These optimizations means that, in the current SpiNNaker tools, profiling shows that processing each synapse in a row takes 21 clock cycles. Because updating each neuron takes 181 cycles we can build the following approximate model of the maximum number of neurons each core can simulate:

(1) $$\delta_{k}={\text{Re}}_{k}-V_{k}$$

Where the simulation time step *dt* = 1 ms and the average input spike rate each neuron receives μ_*input*_ = 8000 × 3 Hz = 24 kHz. Based on the approximate nature of this model and to aid various low-level optimizations 256 neurons are typically simulated on each core.

**Figure 3:**
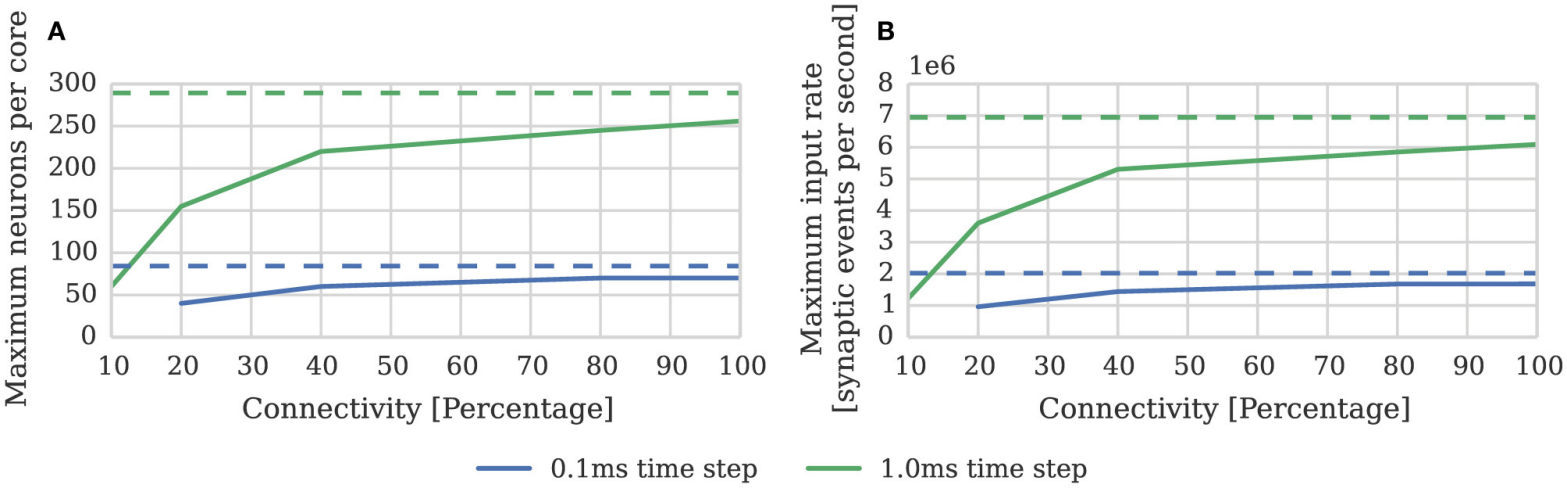
**Static synaptic processing performance of a single SpiNNaker core simulating neurons using simulation time steps of 1 and 0.1 ms**. Each neuron receives 24 kHz of synaptic input from multiple 10 Hz Poisson spike sources, connected with varying degrees of connection sparsity. With a simulation time step of 0.1 ms it was impossible to run simulations with connectivity sparser than 20% in real time. Dotted lines illustrate the performance estimated using Equation (1). **(A)** Performance in terms of the maximum number of these neurons that can be simulated on each core. **(B)** Performance in terms of the raw synaptic event processing performance of each core.

While the connectivity between cortical neurons varies widely, it is always relatively sparse, with recent measurements in the somatosensory cortex of rats (Perin et al., 2011) suggesting that the maximum connection density is around 20%. In order to measure the effect of connection sparsity on the performance of the current simulator we developed a benchmark (similar to that used by Diehl and Cook, 2014) in which a single SpiNNaker core is used to simulate a population of leaky integrate-and-fire neurons. We then stimulate each of these neurons with independent 24 kHz Poisson spike input delivered by multiple 10 Hz sources simulated on additional SpiNNaker cores. Figure 3 compares the performance measured using this benchmark against the estimate provided by Equation (1). As the connectivity becomes sparser each spike source connects to fewer postsynaptic neurons via a shorter synaptic matrix row. Therefore, in order to maintain the same synaptic event processing rate, more input spikes and thus synaptic matrix rows need to be processed. As Figure 3B shows this leads to synaptic input processing performance dropping from 6 × 10^6^ to 3.6 × 10^6^ synaptic events per second as the connection density drops from 100% to the maximum biological connection density of 20%. This occurs because, beyond the 21 clock cycles spent processing each synapse, there is a significant fixed cost: in initiating the DMA transfer of the row; servicing the interrupts raised in response to the arrival of the spike and the completion of the DMA; and setting up the synapse processing loop. Furthermore the only way to counteract the decreasing performance, while maintaining the desired input rate, is to further reduce the number of neurons simulated on each core which further reduces the length of the synaptic matrix rows and thus exacerbates the problem.

In order to increase temporal accuracy (Lagorce et al., 2015) or improve the numerical precision with which the differential equation used to model each neuron are solved (Hopkins and Furber, 2015), it can be necessary to simulate the time-driven components of the SpiNNaker simulation on a shorter time step such as 0.1 ms. Substituting *dt* = 0.1 ms into Equation (1) suggests that, when using this smaller time step, a maximum of 84 neurons can be simulated on each core. However as Figure 3A shows, even with 100% connectivity, the row length is sub-optimal so only 70 neurons can be simulated on each core. Furthermore, as the connectivity becomes sparser, performance drops to the point where, at 10% connectivity, it is impossible to simulate the benchmark in real time.

Lagorce et al. (2015) recently presented an alternative means of simulating neurons with 1 µs temporal accuracy on SpiNNaker using an event-driven neuron model. While some sensory neurons (Gerstner et al., 1996) may require this degree of temporal accuracy, in cortical networks of the type considered in this paper, spike timings are typically only synchronized to within several ms (Riehle et al., 1997). Additionally only a small subset of neuron models can be simulated in an event-driven manner and, if we again consider our model of cortical neurons where each receives an average input rate of 24 kHz, multiple events would need to be processed every 0.1 ms time step by an event-driven model. This would mean that any potential performance advantage would dwindle when compared to a time-driven approach. For these reasons, in the rest of this paper, we will only consider time-driven neural models.

### 2.2. Simulating synaptic plasticity on SpiNNaker

In addition to enabling large-scale simulations with static synapses, the event-driven approach outlined in Section 2.1 can be extended to handle any type of plastic synapse whose state can be updated when a spike arrives based on:

1. The previous state of the synapse.
2. The time at which the last spike was transferred.
3. Information available from the postsynaptic neurons simulated on the local core.

However, when compared to static synapses, simulating plastic synapses is more costly in terms of memory and CPU: both very limited resources on SpiNNaker. Jin et al. (2010) and Diehl and Cook (2014) both developed different solutions to this problem which follow this general event-driven model. In the wider context of distributed computing Morrison et al. (2007) extended their technique for the distributed simulation of static networks to support synaptic plasticity. This approach employed a restricted model of synaptic delay which guarantees that the axonal delay is always shorter than the dendritic delay. Because the dendritic delay is also used to delay backpropagating postsynaptic spikes this restriction means that presynaptic spikes can be processed immediately as no postsynaptic spikes emitted before the axonal delay has elapsed will ever “overtake” the presynaptic spike and hence need to be processed before it. This simplifies the algorithm considerably, reducing CPU and memory usage. The current SpiNNaker simulator combines this simplified delay model and the general approach developed by Diehl and Cook (2014) into Algorithm 1. This algorithm is called whenever the connectivity matrix row associated with an incoming “spike” packet is retrieved from the SDRAM. Each of these rows contains the weights of the synapses that connect the presynaptic neuron to the postsynaptic neurons simulated on the local core (*w*_*ij*_); the time at which the presynaptic neuron last spiked (*t*_lastSpike_) and its state at that time (*s*_*i*_); and the time at which the row was last updated (*t*_lastUpdate_). What data the state (*s*_*i*_) contains depends on the plasticity rule being employed, but as only the presynaptic spike times are available at the synapse, the state often contains one or more low-pass filtered versions of this spike train.

**Algorithm 1:** Algorithmic Implementation of STDP

**function** processRow(*t*, *flush*)
**for each *j* in** *postSynapticNeurons* **do**
*history* ← *getHistoryEntries*(*j*, *t*_lastUpdate_, *t*)

**for each** (*t*_*j*_, *s*_*j*_) **in** *history* **do**
*w*_*ij*_ ← *applyPostSpike*(*w*_*ij*_, *t*_*j*_, *t*_lastSpike_, *s*_*i*_)

**if not** *flush* **then**
(*t*_*j*_, *s*_*j*_) ← *getLastHistoryEntry*(*t*)
*w*_*ij*_ ← *applyPreSpike*(*w*_*ij*_, *t, t*_*j*_, *s*_*j*_)
*addWeightToRingBuffer*(*w*_*ij*_, *j*)

**if not** *flush* **then**
*s*_*i*_ ← *addPreSpike*(*s*_*i*_, *t, t*_lastSpike_)
*t*_lastSpike_ ← *t*

*t*_lastUpdate_ ← *t*

The algorithm begins by looping through each postsynaptic neuron (*j*) in the row and retrieving a list of the times (*t*_*j*_) at which that neuron spiked between *t*_lastUpdate_ and *t*; and its state at that time (*s*_*j*_). In the SpiNNaker implementation these times and states are stored in a fixed-length circular queue located in the tightly-coupled data memory. Whenever a neuron emits a spike a new entry gets added to this structure. As Diehl and Cook (2014) discuss, using a fixed sized data structure instead of the type of dynamic structure described by Morrison et al. (2007) means that postsynaptic spikes can be lost. This will occur if more postsynaptic spikes are emitted between presynaptic spikes than there are buffer entries to store them in. We estimated an optimal size for these buffers based on the lognormal ratio distributions between the cortical firing rates presented by Buzsáki and Mizuseki (2014). From the cumulative density functions of these distributions we found that a 10 entry buffer covers over 90% of the ratio distribution. However, in order to prevent postsynaptic spikes being lost when the pre and postsynaptic neurons have very different firing rates, we developed an additional simple mechanism we call “flushing” to force the processing of these spikes. This mechanism uses one bit in the 32 bit ID associated with each neuron to signify whether the neuron is emitting a “flush” or an actual spike event. To determine when these events should be sent, each postsynaptic neuron tracks its interspike interval (ISI) and, if this is *bufferSize* times longer than the ISI equivalent to the maximum firing rate of the network, a flush event is emitted.

Algorithm 1 continues by looping through each postsynaptic spike and calling the *applyPostSpike* function to apply the effect of the interaction between the postsynaptic spike and the presynaptic spike that occurred at *t*_lastSpike_ to the synapse. If the update was instigated by a presynaptic spike rather than a flush, the *applyPreSpike* function is called to apply the effect of the interaction between the presynaptic spike and the most recent postsynaptic spike to the synapse. Once all events are processed the fully updated weight is added to the input ring buffer. If the update was instigated by a presynaptic spike rather than a flush, after all the synapses are processed, the presynaptic state stored in the header of the row (*s*_*i*_) is updated by calling the *addPreSpike* function; and *t*_lastSpike_ and *t*_lastUpdate_ are set to the current time. If however the update was instigated by a flush event, only *t*_lastUpdate_ is updated to the current time, meaning that the interactions between future postsynaptic events and the last presynaptic spike will continue to be calculated correctly.

We profiled this algorithm in conjunction with *applyPostSpike, applyPreSpike, addPreSpike*, and *addPostSpike* functions that implement pair-based STDP with an additive weight dependence (Song et al., 2000). As the cost of evaluating Algorithm 1 depends on the number of events stored in *history* (*h*) this results in the following approximate model:

(2) $$N_{neurons}=\frac{200\times 10^{6}}{\frac{187}{dt}+(131+31h)\mu_{input}}=49$$

**Figure 4:**
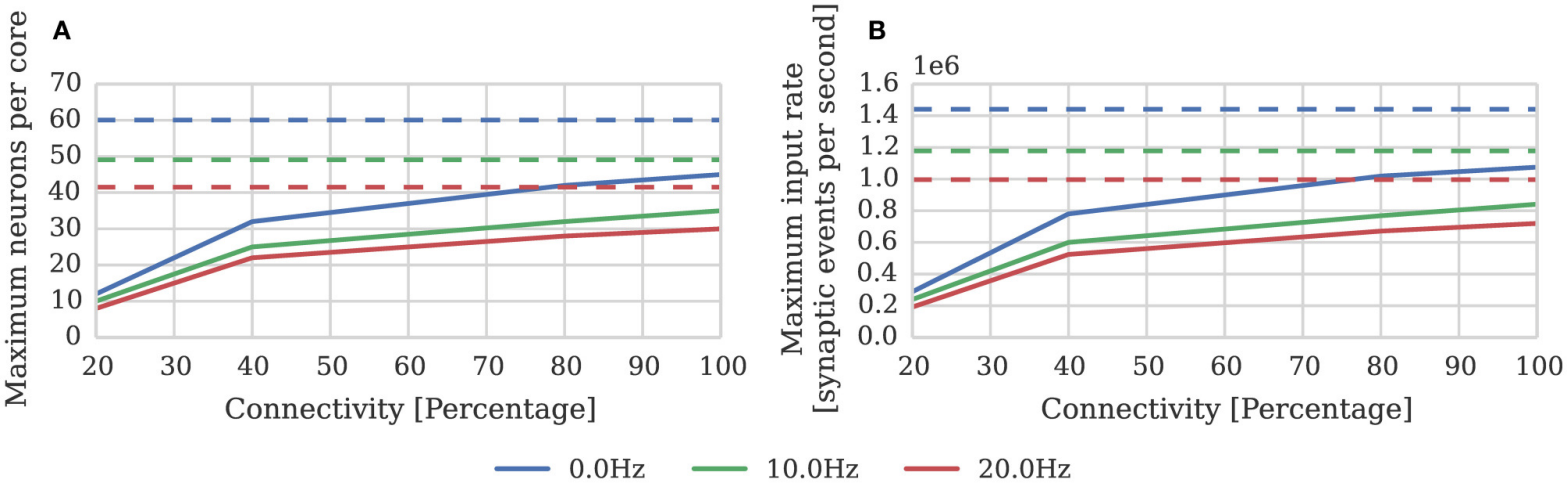
**STDP synaptic processing performance of a single SpiNNaker core simulating neurons with postsynaptic firing rates of 0, 10, and 20 Hz**. Each neuron receives 24 kHz of synaptic input from multiple 10 Hz Poisson spike sources, connected with varying degrees of connection sparsity. Dotted lines illustrate the performance estimated using Equation (2). **(A)** Performance in terms of maximum number of these neurons that can be simulated on each core. **(B)** Performance in terms of raw synaptic event processing performance of each core.

Where again *dt* = 1 m*s* and μ_*input*_ = 24 kHz; and the pre and postsynaptic neurons are firing at approximately the same rate (*h* = 1). In order to measure the effect of connection sparsity and the relative postsynaptic firing rate on actual performance, we extended the benchmark developed in Section 2.1 to use STDP synapses and induced different postsynaptic firing rate by applying a DC input current to the neurons. Figure 4 compares the result of this benchmark against the estimate provided by Equation (2). This benchmark shows that—at just over 1 × 10^6^ synaptic events per second with no postsynaptic activity—the peak performance of our STDP synapses is approximately double the 500 × 10^3^ synaptic events per second performance reported by Diehl and Cook (2014). However, similarly to the static synaptic processing performance discussed in Section 1, the performance drops as low as 191 × 10^3^ synaptic events per second at 20% connectivity due to very short row lengths.

Galluppi et al. (2014) developed a very different approach for simulating synaptic plasticity on SpiNNaker compared to the event-driven approaches we have discussed so far in this section. Galluppi et al. (2014) simulate neurons and their synaptic inputs using the standard approach described in Section 2.1 but use extra cores to simulate plasticity using a more time-driven approach. These “plasticity cores” operate on a relatively slow time step of 128 ms, within which, they read back the entire synaptic matrix row-by-row. Then, based on a record of pre and postsynaptic activity recorded into shared memory during the previous 128 ms by the “neuron core,” the plasticity core updates the synaptic weights. Galluppi et al. (2014) reported that, with the neuron core simulating 100 neurons, this system can perform STDP synaptic processing at rates of up to 1.5 × 10^6^ synaptic events per second per core. However this is based on a benchmark in which the population of neurons being simulated received input from just 195 densely connected, high frequency Poisson inputs meaning that just 195 rows needed to be processed within each 128 ms plasticity time step. If however we consider the model of cortical connectivity described in Section 1 where each neuron has 8000 sparsely connected inputs, even if the connection sparsity is 20%, the synaptic matrix will contain 40,000 rows. If we assume the lowest mean cortical firing rate of around 2 Hz measured by Buzsáki and Mizuseki (2014), each row will contain approximately 20 synapses (based on the 100 neurons per core used in the benchmark) and each row update will have to process an average of 5 postsynaptic events during each 128 ms plasticity time step. As each of the updates performed by the plasticity cores use a trace-based approach similar to Algorithm 1, we can estimate the cost of each resultant update based on the performance model of our own approach. Our model suggests that updating each row will take around 2800 CPU cycles meaning that, as a SpiNNaker core has 256 × 10^5^ clock cycles available within each 128 ms plasticity time step, each plasticity core would be able to update approximately 9100 rows within this time. Therefore the updating of all 40,000 rows would need to be distributed between 5 plasticity cores. This would result in a per-core synaptic processing performance of just 350 × 10^3^ synaptic events per second: only a marginal improvement over the 289 × 10^3^ synaptic events per second achieved by the approach described in this section when simulating neurons with 20% connectivity.

### 2.3. Synapse-centric simulation

In Sections 2.1, 2.2 we identified two main problems with the current approach to mapping large, highly-connected spiking neural networks to SpiNNaker.

1. Synaptic processing performance decreases as connectivity becomes sparser due to shorter synaptic matrix rows over which to amortize the fixed costs of servicing interrupts, initiating the DMA transfer of the synaptic matrix row etc.
2. The only way to reduce the load on a single SpiNNaker core and thus allow neurons with a given synaptic input rate to be simulated in real time is to reduce the number of neurons being simulated on the core, exacerbating the first problem.

**Figure 5:**
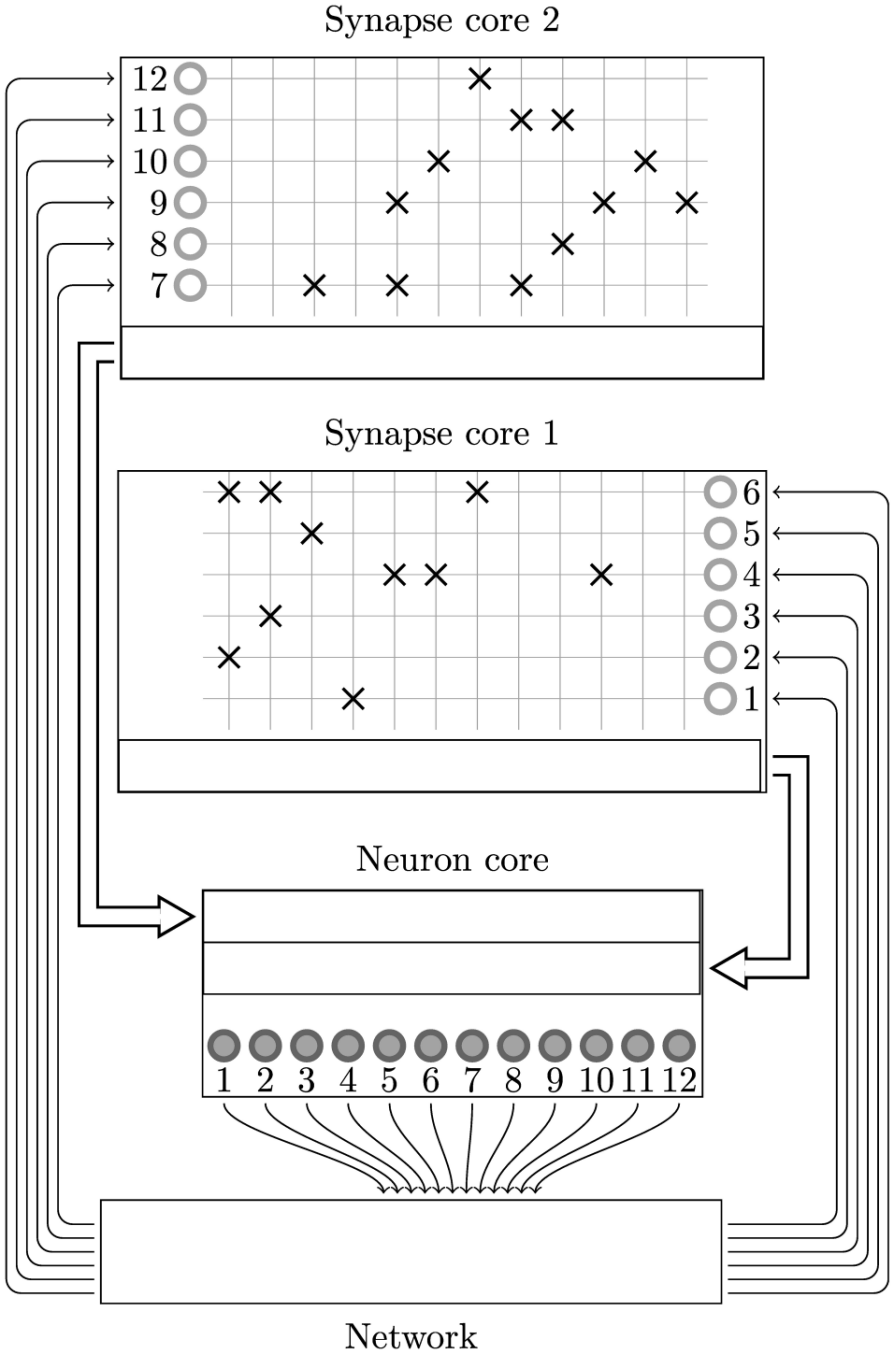
**Synapse-centric mapping of a spiking neural network to SpiNNaker**. The example network used in Figure 2 is distributed between three SpiNNaker cores using the synapse-centric approach. The neuron core is responsible for simulating all 12 neurons (filled circles). The synaptic matrix is split horizontally with the rows associated with the presynaptic neurons (non-filled circles) distributed between two synapse cores. Double arrows indicate how input currents or conductances are transferred from the synapse processors to the neuron processors through shared memory.

In this section we present a novel solution to mapping spiking neural networks with both plastic and static synapses to SpiNNaker which alleviates both of these problems. The key intuition behind this approach is that, if we split the synaptic matrix in a row-wise manner over multiple cores rather than column-wise with the neurons, row lengths can be kept as long as local memory restrictions allow and are unaffected by dividing the synapses amongst multiple cores. As shown in Figure 5 we achieve this by using separate cores to simulate the neurons and their afferent synapses. The afferent synapses associated with the population are split between one or more *synapse processors* based on the following criteria:

1. By synapse type meaning that each synapse processor only needs to have sufficient local memory for a single input ring-buffer and different synaptic plasticity rules can be simulated on separate cores.
2. Postsynaptically (vertically) based on the local memory requirements of the ring-buffer structure and, if the core is simulating plastic synapses, the postsynaptic history structure required for the plasticity algorithm (as discussed in Section 2.2).
3. Presynaptically (horizontally) based on an estimate of the presynaptic processing cost derived from the firing rate of the presynaptic neurons and their connectivity.

The local memory requirements of the input ring-buffer limits each synapse processor to simulating the static synapses associated with 1024 postsynaptic neurons. The extra local memory required for the postsynaptic history structure discussed in Section 2.2 limits synapse processors to simulating the STDP synapses associated with 512 postsynaptic neurons. The synapse processors process the synaptic input using the same event-driven approach outlined in Sections 2.1, 2.2. However, at the beginning of each simulation time step, the synapse processors initiate a DMA transfer to write the input current or conductance accumulated in the ring-buffer (which in the current approach would be passed directly to the neuron model) to a buffer located in the external SDRAM.

As the input currents for each neuron now come from several synapse processors, dedicated *neuron processors* are required to sum each neuron's input currents and update its dynamics. The time-driven simulation of the neurons is split between these neuron processors until memory and real time CPU constraints are met. Without having to also simulate the afferent synapses, each neuron processor can simulate many more neurons than is possible using the current approach. For example 1024 leaky integrate-and-fire neurons with exponential synapses simulating on a 1 ms time step can be simulated on a single core.

At the beginning of each simulation time step each neuron processor initiates a series of DMA reads to fetch the buffers containing the input currents or conductances written by its associated synapse processors. The current or conductance inputs associated with each of the neuron model's receptors are then summed together and passed to the neuron model.

Although the postsynaptic splitting of neurons and synapses can be independent, because the neuron and synapse processors communicate through shared memory buffers only accessible to the 16 cores on the same SpiNNaker chip, this is somewhat restricted. For example if we consider a population of simple leaky-integrate fire neurons—1024 of which can be simulated on a single core—with complex plastic synapses whose local memory requirements mean that they must be split postsynaptically at 256 neurons. If, presynaptically, 4 synapse processors are required to handle the input to these 256 neurons then 17 cores would need to access the same shared memory buffer: more than is present on the SpiNNaker chip. The solution to this problem is to reduce the number of neurons simulated on each neuron processor to 512 meaning that only 9 cores would need to access the same shared memory buffer.

As well as the inputs they receive from other neurons in the network, neurons in cortical models are often kept in an excitable regime by a source of background input. This background input often takes the form of independent Poisson spike trains and, when using the approach discussed in Section 2.1, these are delivered to the neurons using the interconnect network. However the mechanism for providing input to a neuron processor through external memory buffers can be re-used to allow the background input to be delivered from *current input processors* directly to the neuron processors. The current input processors generate a Poisson spike vector every time step, multiply it by a weight vector to convert the spikes into current or conductance values and write the resulting vector to the external memory buffers. The approach described in Section 2.2 would also be difficult to extend to allow populations of neurons to have multiple learning rules on their afferent synapses. This would require the postsynaptic history structure to be extended to include postsynaptic state (*s*_*j*_) for each learning rule adding to its already considerable memory requirements with each additional learning rule. Algorithm 1 would also have to be extended to select the correct learning rule for each synapse and call the appropriate *applyPostSpike* and *applyPreSpike* functions increasing the cost of this, performance critical, algorithm. However supporting multiple learning rules is trivial when using the synapse-centric approach: additional synapse processors can simply be instantiated to simulate each required synapse type.

## 3. Results

### 3.1. Static synaptic processing performance

We profiled the performance of our new static synapse processors and found that the performance has improved over the current approach: down to 15 cycles to process a synapse. This saving comes about because, as each synapse processor only has to process a single type of synapse, the synapse processing loop can be further optimized. Using this figure we can estimate the rate of incoming synaptic events that each synapse processor can handle.

(3) $$\mu_{events}=\frac{200\times 10^{6}}{15}=13\times 10^{6}$$

**Figure 6:**
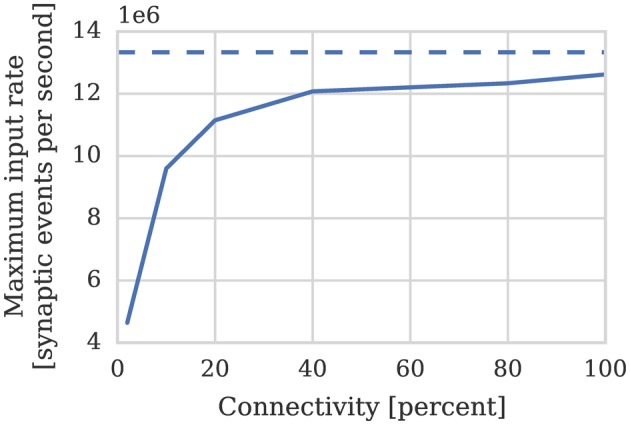
**Performance of a synapse processor simulating the afferent static synapses associated with 1024 neurons**. Each data point represents the maximum input rate (provided by multiple 10 Hz Poisson spike sources) that the core can handle in real time. Dotted line illustrates the performance estimated using Equation (3).

Therefore if we assume—based on our model of cortical connectivity described in Section 1—that each neurons receives 24 kHz of synaptic input we can estimate that 1024 such neurons' afferent synapses could be simulated using 2 synapse processors. In order to verify these results we repeated the benchmark described in Section 2.1 on a population of 1024 neurons mapped to one neuron processor and one synapse processor using our new synapse-centric approach. Figure 6 shows that the peak performance of the synapse processor is indeed almost 13 × 10^6^ synaptic events per second although this reduces significantly with sparser connectivity. However, because the number of postsynaptic neurons does not need to be reduced until all of the afferent synapses can be simulated on a single core and because the length of a row representing the same connectivity is 4× longer than it would be when using the current approach, this effect is significantly less pronounced. On this basis, just 2 synapse processors can handle 100% connectivity and 3 can handle the same situation with 10% connectivity. Therefore, including the neuron processor, 341 neurons can be simulated per core at 100% connectivity and 256 per core at 10% connectivity: a significant improvement over the 256 and 155 achieved using the current approach.

**Figure 7:**
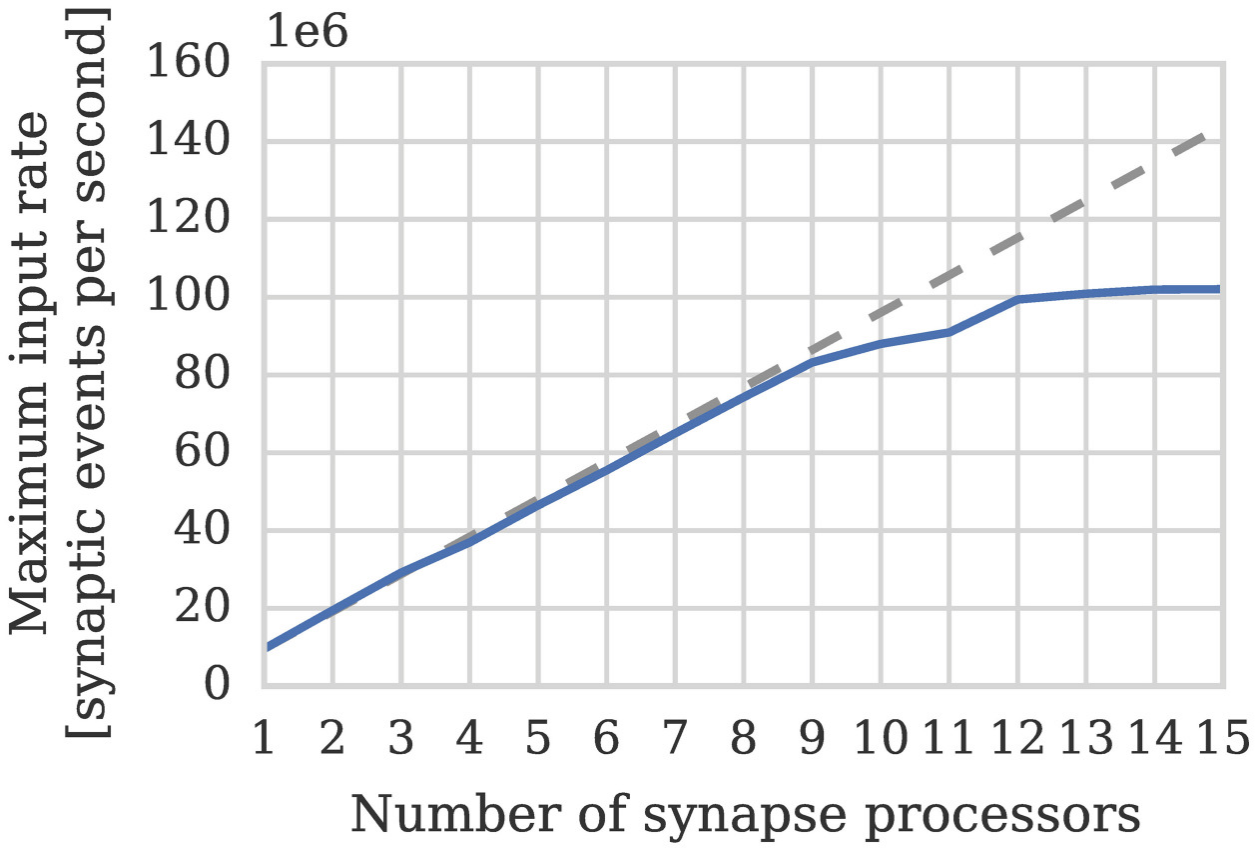
**Performance of SpiNNaker chip containing one neuron processor simulating population of 512 neurons and increasing numbers of synapse processor simulating the afferent static synapses associated with the population**. Each data point represents the maximum input rate (provided by multiple 10 Hz Poisson spike sources) that the core can handle in real time. 20% connection sparsity is used for all data points. The dotted line shows the linear scaling of the performance with one synapse processor.

**Figure 8:**
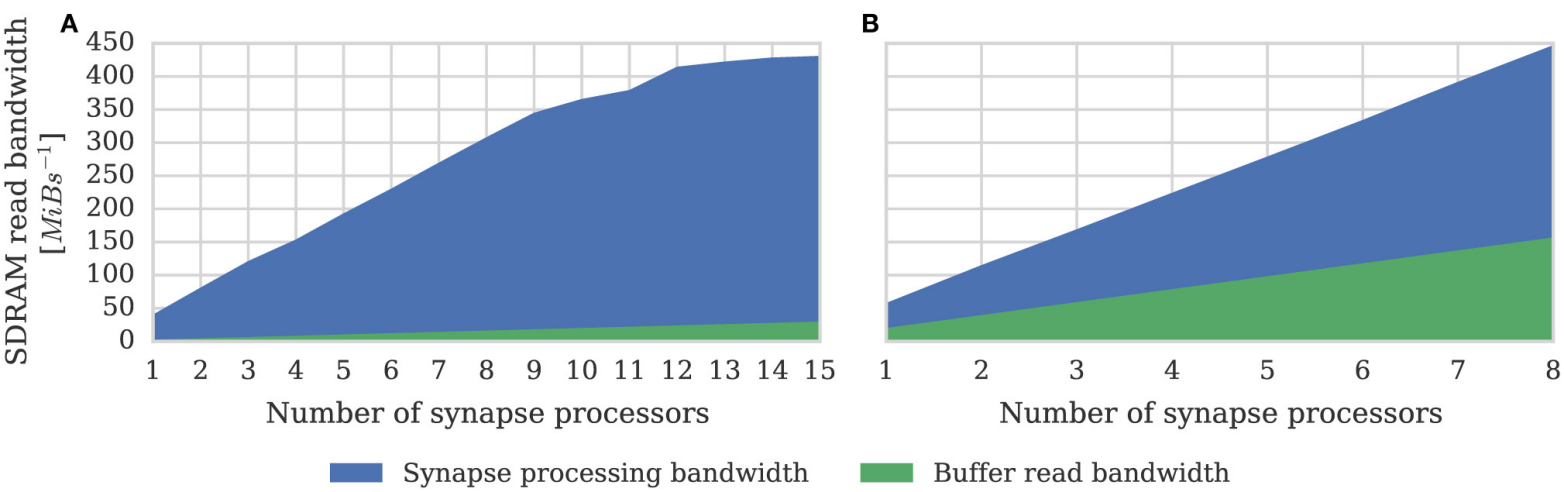
**External memory read bandwidth used by SpiNNaker chip containing increasing numbers of synapse processors simulating the afferent static synapses associated with 512 neurons**. Colors indicate how much of this bandwidth is used transferring synaptic matrix rows and how much for transferring input currents to the neuron processor(s). 20% connection sparsity is used for all data points. **(A)** With a simulation time step of 1 ms where the 512 neurons are simulated on a single neuron processor. **(B)** With a simulation time step of 0.1 ms where the 512 neurons are simulated across 8 neuron processors.

One potential downside of the synapse-centric approach is that transferring input via SDRAM from the synapse to the neuron processors every simulation time step requires extra external memory bandwidth. In order to determine whether this affects the scaling of our synapse-centric approach, we extended our benchmark to use multiple synapse processors with the inputs divided evenly between them. Figure 7 shows that with up to 9 synapse processors, synaptic processing performance grows linearly as more synapse processors are added, with each additional synapse processor adding approximately 10 × 10^6^ synaptic events per second to the total performance. However the performance plateaus with 12 synapse processors delivering a synaptic processing performance of around 100 × 10^6^ synaptic events per second. Fetching the synaptic matrix rows required by a single synapse processor requires approximately 40 MiB s^−1^ of external memory bandwidth and transferring the input currents associated with 512 neurons every 1 ms simulation time step requires approximately another 2 MiB s^−1^. Figure 8A shows the external memory read bandwidth usage in our benchmark and—similarly to the performance shown in Figure 7—this increases linearly with up to 9 synapses processors and plateaus at 420 MiB s^−1^. If we reduce the simulation time step to 0.1 ms the bandwidth required to transfer the input currents from each synapse processor increases to 20 MiB s^−1^. Figure 8B shows the results of repeating our benchmark on a 0.1 ms simulation time step with 8 neuron processors and up to 8 synapse processors. Because of the increased bandwidth required to transfer input currents every 0.1 ms, this configuration has a significantly higher peak bandwidth of 450 MiB s^−1^, but shows no sign of the performance plateauing.

In order to illustrate the advantages of our new simulator in the context of a more realistic network we ran several simulations of the network developed by Vogels and Abbott (2005). This network was designed as a medium for experimentation into signal propagation through cortical networks, but has subsequently been widely used as a benchmark (Brette et al., 2007). The network consists of 10,000 integrate-and-fire neurons, split between an excitatory population of 8000 cells and an inhibitory population of 2000 cells. In order to be representative of long-range cortical connectivity these populations are randomly connected with a very low connection probability of 2%. Table 1 shows that if we run a 500 ms simulation of this network on SpiNNaker, using either 1 or 0.1 ms time steps, our new approach requires fewer cores than the current approach. However, due to its small size and sparse connectivity, each neuron in this network only receives 200 synaptic inputs: far below the degree of connectivity seen in the cortex and the performance limits of our synapse processors. Therefore, we increased the connection density of the network to 10% (the highest density at which Vogels and Abbott (2005) suggests their results hold) and increased the total number of neurons to 80,000 so that each neuron in the network receives 8000 inputs. Because the neurons in this network have both inhibitory and excitatory synapse, in our synapse-centric approach, they are simulated on separate synapse processors. Therefore, an extra synapse processor—beyond the 3 we previously calculated—is required to simulate the synapses associated with each 1024 neurons. As discussed in Section 2.3 each neuron processor can simulate up to 1024 leaky-integrate fire neurons. However processing this many neurons leaves insufficient time within a simulation time step to process the input from 4 synapse processors. Therefore, we reduced the number of neurons simulated on each neuron processor to 512, resulting in an average of 170 neurons being simulated on each core. This is a significant improvement over the 60 neurons per core our benchmark–shown in Figure 3–suggests the standard approach can achieve.

**Table 1:** **Simulations of the Vogels Abbott benchmark networks on SpiNNaker using synapse-centric and standard approaches**.

**Number of neurons**	**Connectivity [%]**	**Simulator**	**Simulation time step [ms]**	**Number of cores**			**Neurons per core**
				**Neuron**	**Synapse**	**Total**	
10,000	2	Standard	1.0	40		40	250
		Synapse-centric	1.0	10	20	30	333
10,000	2	Standard	0.1	157		157	64
		Synapse-centric	0.1	99	26	125	80
80,000	10	Synapse-centric	1.0	157	314	471	170

### 3.2. Plastic synaptic processing performance

We profiled the performance of one of our synapse processor cores simulating synapses with pair-based STDP and an additive weight dependence (Song et al., 2000). Similarly to the static synapse processors, due to the optimizations made possible as only a single type of synapse is simulated on each synapse processor, the performance was somewhat improved over that of the current approach. Based on the model obtained through this profiling we can estimate the rate of incoming synaptic events that each STDP synapse processor can handle.

(4) $$\mu_{events}=\frac{200\times 10^{6}}{107+30h}=\;1.4\times 10^{6}$$

**Figure 9:**
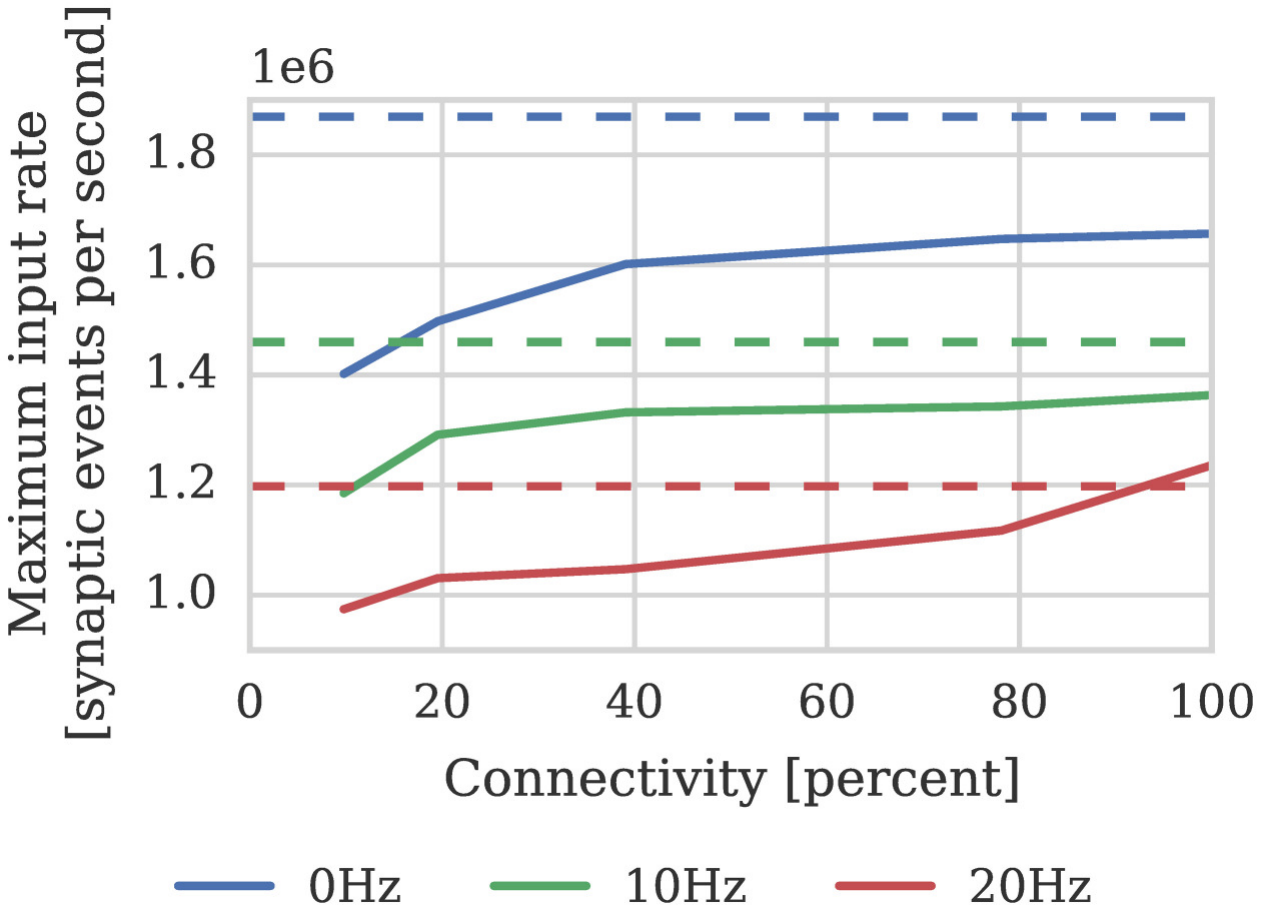
**Performance of a synapse processor simulating the afferent STDP synapses associated with 512 neurons**. Each data point represents the maximum input rate (delivered by multiple 10 Hz Poisson spike sources) that the synapse processor can handle in real time with varying levels of connectivity when postsynaptic neurons are firing at 0, 10, or 20 Hz. Dotted lines illustrate the performance estimated using Equation (4).

Where the pre and postsynaptic neurons are firing at approximately the same rate (*h* = 1). As discussed in Section 2.3 the local memory requirements of the postsynaptic history structure mean that each STDP synapse processor can simulate the afferent synapses associated with 512 neurons. Therefore, if we divide the total estimated performance between 512 neurons and again use our model of cortical connectivity we can estimate that the afferent synapses associated with our 512 neurons can be simulated using 9 synapse processors. In order to verify this performance we repeated the STDP benchmark described in Section 2.2 using a population of 512 neurons mapped to one neuron processor and one synapse processor using our new synapse-centric approach. The results of this benchmark are presented in Figure 9 and show that the peak performance is indeed nearly 1.4 × 10^6^ synaptic events per second. Because processing an STDP synapse is significantly more costly than processing a static synapse, the fixed cost of processing a row is amortized over fewer synapses, meaning that 10 STDP synapse processors are sufficient to deliver our model of cortical connectivity down to just over 10% connection sparsity. Therefore taking into account the core used by the neuron processor, with 20% connectivity, 46 neurons can be simulated per core: more than 4× the number possible when using the current approach.

Knight et al. (2016) demonstrated how the spiking BCPNN learning rule (Tully et al., 2014) could be implemented efficiently on SpiNNaker within the algorithm outlined in Section 2.2. Knight et al. (2016) then showed how this learning rule could be used to learn temporal sequences of neural activity within a modular attractor network. For more details on the biological underpinnings of this network and further examples of its function see Tully et al. (2016). This network was based on a cortical microcircuit model developed by Lundqvist et al. (2006) consisting of a number of *hypercolumns* arranged in a grid. Each hypercolumn consists of 250 inhibitory and 1000 excitatory cells evenly divided between 10 minicolumns. While this was the largest plastic neural network ever to be simulated on neuromorphic hardware, the training process was hampered by the inability of the approach described in Section 2.2 to simulate neurons with different learning rules on their afferent synapses. This limitation meant that separate networks had to be simulated to train the AMPA and NMDA synapses, the learned weights downloaded, combined together and finally re-uploaded to the SpiNNaker machine for testing. This model also had several features that placed high demands on the local memory available to each core. Firstly BCPNN requires 32 bit of state to be stored with each event in the postsynaptic history structure rather than the 16 bit required by STDP synapses meaning that a 10 entry postsynaptic history requires an extra 20 B of local memory for each neuron. Additionally the model uses three synapse types (AMPA, NMDA, and GABA)—each of which require a separate input ring-buffer—and each neuron in the network also has several extra parameters used to configure a simple spike frequency adaption mechanism (Liu and Wang, 2001). These factors conspired to reduce the local memory available and, when combined with the high cost of simulating BCPNN synapses, meant that, although each neuron in the model only had 4000 inputs, only 75 neurons could be simulated on each core and the network could only be run at 0.5× real time.

**Figure 10:**
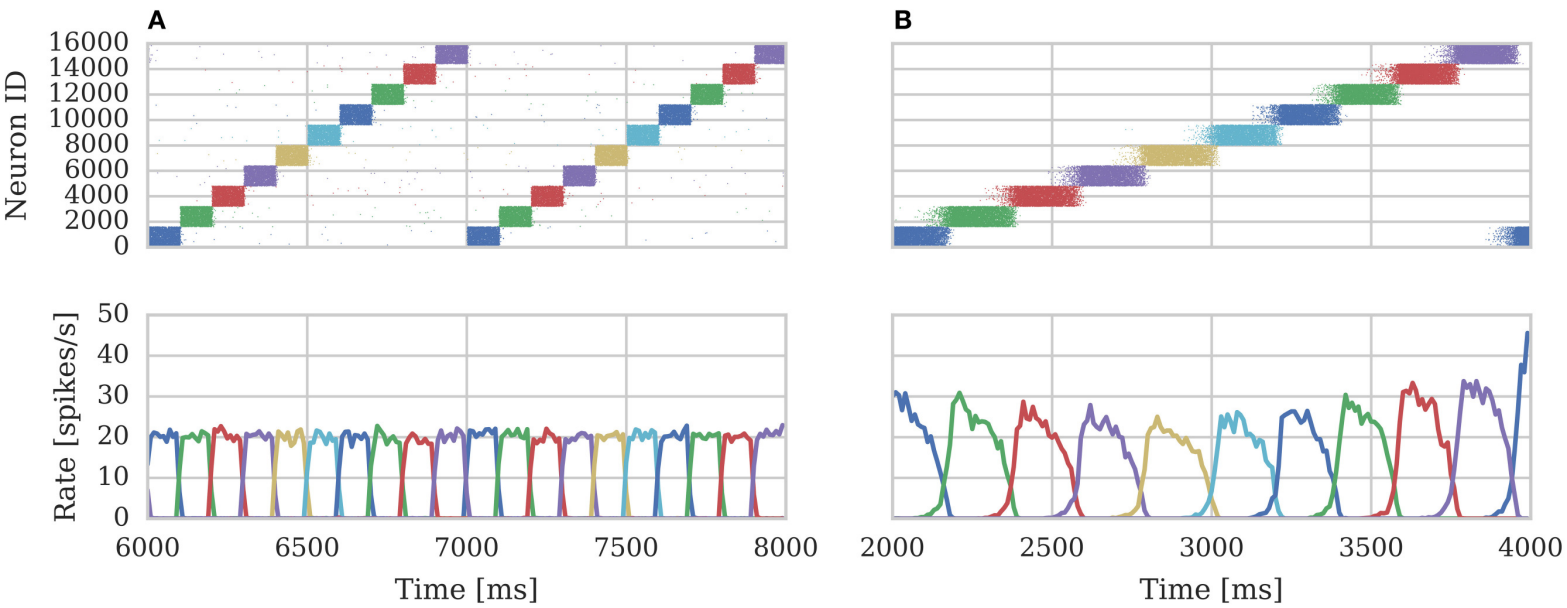
**Spike rasters of excitatory cells and the average firing rate within each minicolumn during subset of (A) training and (B) replaying of temporal sequences of minicolumn activation in 16 hypercolumn modular attractor network**.

**Figure 11:**
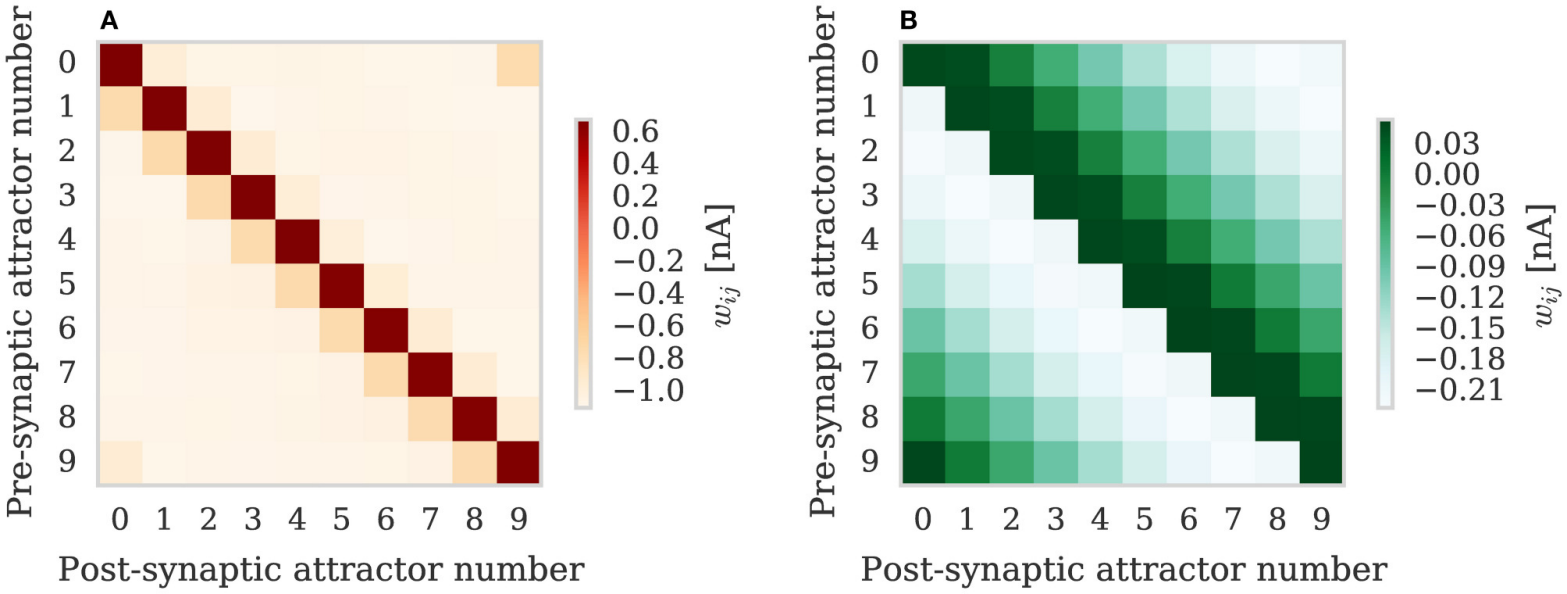
**Average weights learned between minicolumns after training modular attractor network using BCPNN learning rule**. **(A)** AMPA weights. **(B)** NMDA weights.

We repeated the training regime performed by Knight et al. (2016) and trained each hypercolumn with a repeating temporal sequence of minicolumn activations (a subset of this training regime is shown in Figure 10A). Using our new approach we trained both the AMPA and NMDA plastic synapses simultaneously on separate synapse processors each with different BCPNN configurations. The AMPA synapses are trained using spiking BCPNN configured to detect correlations within a short, symmetrical time window resulting in the learned connectivity shown in Figure 11A which acts to sharpen and stabilize activity within a single minicolumn. However the spiking BCPNN learning rule used to learn NMDA connectivity is configured to detect correlations in a much longer, asymmetrical time window resulting in the connectivity shown in Figure 11B. When combined with the spike frequency adaption mechanism, this asymmetrical connectivity acts to enable sequence transitions, allowing learned sequences of minicolumn activation to be replayed as shown in Figure 10B when plasticity is turned off and the first element of the sequence is stimulated.

Table 2 summarizes the results of simulations of this modular attractor network with 4, 9, and 16 hypercolumns using both the synapse-centric and standard approaches. While the synapse-centric approach requires more cores in all but the 4 hypercolumn configuration, it allows the network to be simulated in real time in all configurations. The standard approach can only simulate the network in real time with 4 hypercolumns and would require the neural populations to be further sub-divided to achieve real time performance at larger scales. Furthermore the comparison is a somewhat unfair as, when using the standard approach, only one synapse type is learned at once meaning that, during the training phase when plasticity is enabled, each core only needs to be capable of handling half the rate of incoming synaptic events.

**Table 2:** **SpiNNaker simulations of the BCPNN modular attractor network at varying scales using synapse-centric and standard approaches**.

**Number of hypercolumns**	**Simulator**	**Real time**	**Heterogeneous learning rules**	**Number of cores**
4	Standard	✓	✗	88
	Synapse-centric	✓	✓	68
9	Standard	✗	✗	198
	Synapse-centric	✓	✓	252
16	Standard	✗	✗	352
	Synapse-centric	✓	✓	576

## 4. Discussion

The contribution of this study is threefold. Firstly we present an in-depth analysis of the performance of the current SpiNNaker simulator in the context of highly-connected cortical models. Secondly we present a novel approach for mapping the simulation of such models to SpiNNaker and show how this can significantly increase the size of network that can be simulated on a given SpiNNaker machine. Thirdly we show that, not only does our approach offer even more significant efficiency savings when simulating cortical models with plastic synapses, but it also enables the simulation of neurons with multiple types of plastic synapse. Finally, in this section, we will discuss the performance of our novel approach, some of the new possibilities it enables and its applicability to current and future hardware platforms.

### 4.1. Synapse-centric simulation

In Section 3.1 we analyze the peak synaptic processing performance of an entire SpiNNaker chip using our new synapse-centric approach. We find that with up to 9 synapse processors running on each SpiNNaker chip, performance scales linearly, but plateaus with 12 synapse processors at around 100 × 10^6^ synaptic events per second. Maintaining this throughput requires 420 MiB s^−1^ of external memory read bandwidth. This is significantly lower than the peak external memory read bandwidth of 600 MiB s^−1^ measured by Painkras et al. (2013). Therefore, we believe that this plateau occurs when contention for access to the external memory increases the duration of each DMA transfer to the point where double-buffering can no longer hide the external memory latency. However if we reduce the simulation time step to 0.1 ms—requiring input currents to be transferred from the synapse processors to the neuron processors 10× more frequently—450 MiB s^−1^ of external memory read bandwidth can be obtained. This supports the view that the plateauing of performance is not due to the memory bandwidth being saturated. Furthermore, by simulating a more realistic network of 80,000 neurons each with 8000 sparsely connected inputs, we demonstrate that 8 synapse processors and 4 neuron processors running on a SpiNNaker chip is likely to be a more typical configuration for simulating cortical networks with static synapses. This configuration is well within the region where Figures 7, 8 show linear performance scaling and leaves 4 cores free to provide additional background noise or stimuli to the neurons.

In Section 3.2 we analyze the performance of pair-based STDP synapses with an additive weight dependence (Song et al., 2000) and find that they are between 6.5× and 10× more costly to simulate than static synapses. This reduction in performance compared to static synapse processors corresponds to similarly reductions in memory read bandwidth requirements meaning that the static network represents the worst case in terms of external memory bandwidth requirements.

### 4.2. Multi-compartmental neural simulation

As we demonstrated in Section 3.2 our approach enables neurons with heterogeneous plastic synapses to be simulated. However neurons in the cortex have many more degrees of heterogeneity particularly in the morphology and complexity of their dendritic trees (Elston, 2003). This dendritic complexity is mirrored in the hierarchical organization of cortical areas (Riesenhuber and Poggio, 1999) and there is mounting evidence to suggest that single dendritic branches rather than individual neurons may, in fact, be the brain's fundamental functional units (Branco and Häusser, 2010).

However the type of point neuron models—which have thus far been used in SpiNNaker simulations—do not model the affects of this structural complexity. Typically models with more complex dendritic trees are simulated by splitting the dendritic tree into *compartments* within which the membrane voltage is assumed to be constant. Each of these compartments is then simulated numerically with ohmic channels being used to exchange current with neighboring compartments (Dayan and Abbott, 2001, p. 217). Potentially our new simulator could provide the basis for mapping such a model onto SpiNNaker by adding *dendritic compartment processors*. The dendritic compartment processors would, like the current neuron processors, receive synaptic input from synapse processors through memory buffers. Additionally they would also receive membrane voltages from neighboring neuron and dendritic compartment processors through additional memory buffers. During each simulation time step the dendritic compartment processors would update the state of their dendritic compartment and write its membrane voltages to a memory buffer.

### 4.3. Design of future neuromorphic hardware

The synapse-centric simulator we present in Section 2.3 transfers input currents or conductances between cores via buffers in external memory because SpiNNaker provides no other means of bulk on-chip communications. The SpiNNaker communications NoC is designed for the low latency transfer of the small packets used to represent spikes rather than bulk transfers and the system NoC does not support direct core-to-core communications (Plana et al., 2007).

In Section 3.1 we demonstrate that the extra external memory bandwidth this requires is unlikely to saturate the memory bandwidth of the current SpiNNaker system. However, while the basic computational units of future, software-programmable neuromorphic systems are likely to be somewhat more powerful than those used by SpiNNaker, such systems are likely to obtain improved performance largely through taking advantage of smaller process sizes in order to integrate more cores into each chip-multiprocessor (CMP) (Olukotun et al., 1996). Furthermore, the gap in performance between DRAM and CPUs has only increased since the SpiNNaker architecture was originally conceived, meaning that providing sufficient external memory bandwidth for a CMP with a larger number of cores is likely to present a significant challenge. These architectural pressures act to make external memory bandwidth more precious, meaning that the extra demands of the synapse-centric approach may be unacceptable. It is also likely that future systems will target the simulation of more complex neuron models, perhaps even the type of multi-compartmental model discussed in Section 4.2. As Hopkins and Furber (2015) discussed, in order to accurately simulate more complex models, smaller simulation time steps are likely to be necessary but, as Figure 8B shows, the increased frequency at which buffers have to be exchanged further exacerbates the problem.

Beyond our synapse-centric approach, the ability to share data between cores without sacrificing external memory bandwidth allows any application to extract a second level of finer-grained parallelism than message passing alone allows. This capability could be incorporated into the design of future systems by employing a NoC architecture that allows cores to access other cores' local memory, either directly or via a DMA controller. This would have additional benefits for the system's fault tolerance as it would allow the contents of a crashed core's local memory to be transferred to another core allowing it to continue from the same state.

### 4.4. General applicability of the approach

Typically, when large distributed computer systems are used for simulating spiking neural networks (Morrison et al., 2005; Kunkel et al., 2012), the simulations are run as batch processes and the primary concern has been to minimize their memory footprint so that the large networks can fit in the systems memory. However, more recently, these distributed systems have been used to run simulations in a closed-loop with virtual robotic environments (Weidel et al., 2016): a situation in which running in real time, or more generally, reducing run time becomes more important. Knight et al. (2016) reported that, in order to approximately half the simulation time of the modular attractor network discussed in Section 3.2, it had to be distributed between more than 4× as many cores of a Cray-XC30 supercomputer (Cray, 2013) resulting in each core only simulating around 100 neurons. While large distributed computer systems use a wide variety of MPI interconnect technologies, the bandwidth they deliver generally drops as message size reduces (Liu et al., 2003), meaning that in the situation where each core is only simulating 100 neurons packet size is likely to be sub-optimal. However, if a variant of our synapse-centric approach was used on these systems, synapses and neurons could be distributed between the cores of each shared-memory compute node and the larger number of neurons simulated on each neuron core would be able to employ MPI to transmit spikes more efficiently. Additionally because—unlike the SpiNNaker communications NoC—MPI is not a multicast technology reducing the postsynaptic splitting of synaptic matrices would reduce the number of cores spikes need to be sent to.

## Author contributions

JK developed the synapse-centric simulator and performed the experiments. JK and SF wrote the paper.

## Funding

The design and construction of SpiNNaker was funded by EPSRC (the UK Engineering and Physical Sciences Research Council) under grants EP/G015740/1 and EP/G015775/1. The research was supported by the European Union under grant number FP7-604102 (HBP) and by the European Research Council under the European Union's Seventh Framework Programme (FP7/2007-2013)/ERC grant agreement 320689. JK is supported by a Kilburn Studentship from the School of Computer Science at The University of Manchester and a President's Doctoral Scholar Award.

### Conflict of interest statement

The authors declare that the research was conducted in the absence of any commercial or financial relationships that could be construed as a potential conflict of interest.
